# Factors associated with pulmonary impairment in HIV-infected South African adults

**DOI:** 10.1371/journal.pone.0184530

**Published:** 2017-09-13

**Authors:** Akshay N. Gupte, Michelle L. Wong, Reginah Msandiwa, Grace L. Barnes, Jonathan Golub, Richard E. Chaisson, Christopher J. Hoffmann, Neil A. Martinson

**Affiliations:** 1 Johns Hopkins Bloomberg School of Public Health, Baltimore, Maryland, United States of America; 2 Center for Clinical Global Health Education (CCGHE), Johns Hopkins University School of Medicine, Baltimore, Maryland, United States of America; 3 Chris Hani Baragwanath Academic Hospital and Faculty of Health Sciences, University of the Witwatersrand, Johannesburg, South Africa; 4 Perinatal HIV Research Unit (PHRU), MRC Soweto Matlosana Collaborating Centre for HIV/AIDS and TB, University of the Witwatersrand, Johannesburg, South Africa; 5 Center for Tuberculosis Research, Johns Hopkins University School of Medicine, Baltimore, Maryland, United States of America; Katholieke Universiteit Leuven Rega Institute for Medical Research, BELGIUM

## Abstract

**Background:**

HIV-infected individuals have increased risk of developing obstructive lung disease (OLD). Studies from developed countries report high viral load, low CD4 counts, and anti-retroviral therapy (ART) to be associated with OLD; but these findings may not be generalizable to populations in resource-limited settings.

**Methods:**

We conducted a prospective cohort study of lung function in 730 HIV-infected black South African adults. Pre-bronchodilator spirometry was performed at enrollment and repeated annually for three years. Logistic regression models were used to identify factors associated with OLD, defined as FEV1/FVC<0.70, at enrollment. Excess annual declines in FEV1 and FVC were modelled as the product-term of follow-up time and exposures using random effects regression.

**Results:**

Median (IQR) age at enrollment was 36 (32–41) years, 85% were female and 30% ever-smoked with a median (IQR) exposure of 3 (1–6) pack-years. Median (IQR) CD4 count and viral load at enrollment were 372 (261–518) cells/mm^3^ and 2655 (91–13,548) copies/mL respectively. Overall, 25% were receiving ART at enrollment, 16% of whom reported at least 6 months of ART receipt. OLD was found in 35 (5%) at enrollment. Increasing age (aOR = 2.08 per 10-years [95%CI 1.22–3.57], p = 0.007), current smoking (aOR = 3.55 [95%CI 1.20–10.53], p = 0.02), and CRP (aOR = 1.01 per unit-increase [95%CI 1.00–1.03], p = 0.04) were significantly associated with OLD at enrollment; while increasing CD4 count (aOR = 1.02 per-100 cells/mm^3^ [95%CI 0.85–1.22], p = 0.82), viral load (aOR = 0.67 per log-increase [95%CI 0.43–1.10], p = 0.12) and receipt of ART (aOR = 0.57 [95%CI 0.18–1.75], p = 0.32) were not. The median (IQR) follow-up time was 18 (12–24) months. Participants with a history of tuberculosis (TB) had a 35 mL (95%CI 2–68, p = 0.03) and 57 mL (95%CI 19–96, p = 0.003) per year excess loss of FEV1 and FVC respectively.

**Conclusion:**

Prevalent OLD was associated with older age, current smoking and higher CRP levels, but not CD4 counts and ART, in HIV-infected South African adults. Better understanding of the long-term effects of TB, smoking and inflammation on lung function in HIV-infected populations is urgently needed.

## Introduction

The WHO estimates 37 million people are infected with HIV, with sub-Saharan Africa accounting for nearly 70% of the global burden [1]. Widespread use of anti-retroviral therapy (ART) has led to dramatic declines in opportunistic infections and increased life expectancy. However, the burden of non-communicable diseases, such as chronic obstructive pulmonary disease (COPD) and asthma, is increasing in people living with HIV [2–4]. Cross-sectional studies have found a higher prevalence and up to a 10-fold increased odds of obstructive lung disease (OLD) in adults with HIV infection compared to those without [5, 6]. Furthermore, the incidence of COPD was significantly higher in HIV-infected compared to—uninfected individuals irrespective of age, suggesting temporality of the HIV-OLD association [7].

Although HIV is an independent risk factor for OLD, key factors associated with pulmonary impairment in HIV-infected individuals are unclear. For instance, the AIDS Linked to the Intravenous Experience (ALIVE) cohort, comprising of HIV-infected and -uninfected injection drug users (IDUs) in Baltimore, USA, found high viral load, but not CD4 cell counts or heavy smoking, to be associated with OLD and excess declines in lung function [8, 9]. Conversely, lower CD4 cell counts, heavy smoking and IDU were associated with higher odds of COPD in the US Veterans Aging Cohort (VA) [5]. In the longitudinal follow up of the VA cohort, higher viral load was associated with incident COPD; however, this association lost significance after adjusting for smoking [7]. Furthermore, recent studies have reported increased odds of OLD in ART experienced compared to -naïve individuals [10, 11]; however, these findings were not replicated in subsequent studies [8, 12, 13].

While current studies have considerably advanced our understanding of the HIV-OLD association, HIV-infected individuals at greatest risk of pulmonary impairment are yet to be identified. Furthermore, studies investigating OLD and excess lung function decline in those infected with HIV were conducted in unique populations (e.g., US veterans, IDUs) from high-income settings, or in populations with high levels of smoking and/or IDU, which may confound the association between markers of HIV disease and pulmonary impairment. The objective of our study was to identify factors associated with OLD and excess lung function decline among HIV-infected adults in South Africa; where nearly 20% of the adult population is living with HIV [14]. Addressing these knowledge gaps could improve care of HIV-infected individuals living in the heart of the HIV epidemic through better understanding of the HIV-OLD association and targeted interventions.

## Material and methods

### Study design and population

We established a prospective cohort of 755 HIV-infected adults (>18 years) at the Perinatal HIV Research Unit (PHRU) in Soweto, South Africa from 2008 to 2011. Participants were enrolled from two sources: 1) a recently completed phase-3 randomized controlled trial of three novel tuberculosis (TB) preventive regimens in *Mycobacterium tuberculosis* infected and ART naïve HIV-infected adults with CD4 counts ≥ 200 cells/mm^3^ (NCT00057122) [15], and 2) an outpatient HIV clinic located at the Chris Hani Baragwanath Academic Hospital [16, 17]. Pregnant or breast-feeding women and participants with a life-threatening medical condition were excluded. Additionally, we excluded six participants of white or Indian ethnicity from our analysis as they accounted for less than 1% of our cohort.

## Study procedures

Enrolled participants were followed for three years. Clinical evaluations, CD4 cell count, viral load and plasma C-reactive protein (CRP) assessments were performed at enrollment and every six months thereafter. History of smoking, TB, pneumocystis pneumonia (PCP), second-hand smoking (SHS), exposure to biomass fuels (burning of wood, animal-dung, coal or paraffin for cooking or heating purposes), and occupation were obtained by self-report. Spirometry without bronchodilators was performed using an ultrasound spirometer (KoKo Spirometer by nSPIRE Health, Colorado, USA) at enrollment and every 12 months thereafter. All spirometry measurements were conducted by a qualified pulmonary function technologist according to ATS/ERS standards [18], and the curves assessed by a trained pulmonologist. Unacceptable spirometry curves were excluded from the analysis. All study participants signed written informed consent in their native language and the protocol was approved by the Institutional Review Boards of Johns Hopkins Medicine and the University of the Witwatersrand.

### Analysis

Baseline characteristics between groups were compared using the t-test and Wilcoxon rank sum test for normally and non-normally distributed continuous data respectively. Categorical data were compared by the X^2^ or Fischer’s exact tests. Percent predicted values of FEV1, FVC and FEV1 to FVC ratio were calculated using reference equations derived from an adult population of black African ethnicity from Johannesburg, South Africa [19].

Univariate and multivariate logistic regression models were constructed to measure associations between participant characteristics and OLD, defined as FEV1 to FVC ratio less than 0.70, at enrollment [20]. Independent variables were modelled continuously (age, BMI, tobacco pack-years, CD4 cell count, log-transformed viral load, CRP level), at relevant increments (age per-10 years, CD4 count per-100 cells/mm^3^) and categorically at relevant thresholds (BMI at 18.5 and 25; pack-years at 10; CD4 count at 200, 350 and 500 cells/mm^3^; viral load at 50 [limit of detection], 2655 [median] and 13,548 [75^th^ percentile] copies/mL; CRP at 1, 3 and 9 mg/L). Smoking was modelled as a dichotomous (ever vs never) and categorical (never vs former or current) variable. Ever smoking was defined as having consumed at least 100 cigarettes and current smokers were defined as those who continued to smoke on a regular basis. SHS was defined as having ever lived with a regular smoker since 14 years of age. ART and TB were modelled as dichotomous (ever vs never) variables. The multivariate model was constructed based on literature review and exploratory data analysis, and included factors known to be associated with OLD (age, smoking, TB), markers of HIV disease (CD4 cell count, viral load, ART), variables that were significant at the p<0.10 level in univariate analysis (CRP level, SHS) and potential confounders (sex, education, BMI). We additionally performed separate analysis for OLD defined as FEV1 to FVC ratio less than the 5^th^ percentile of expected for age, sex and height using previously described reference equations [19].

Next, we measured excess change (mL/year) in FEV1 and FVC, modelled as the product-term of independent variables and follow-up time, using random effects linear regression. Participant characteristics were modelled as baseline (age, sex, BMI, education, ever smoking, ART, TB, PCP) and time-varying (current smoking, CD4 cell count, viral load, CRP level) exposures. CD4 cell counts, viral load and CRP levels were modelled as dichotomous variables at clinically relevant thresholds. Multivariate models were constructed based on literature review and exploratory data analysis, and were adjusted for baseline FEV1 or FVC as relevant. Additionally, assuming the underlying biological mechanisms of lung injury to be chronic in nature, we substituted time-updated CD4 and viral load data with those obtained from 6 months prior to a spirometry evaluation in separate analyses. Finally, we performed sensitivity analysis by excluding participants who were lost to follow-up, died or otherwise did not complete at least three follow-up spirometry evaluations.

All multivariate models were tested for collinearity using the variance inflation factor (VIF), and variables with VIFs of 2 or higher were reassessed. Statistical significance was determined at p<0.05. Analyses were done in STATA V.13.0 (StataCorp, Texas).

## Results

### Cohort description at enrollment

**Fig 1:**
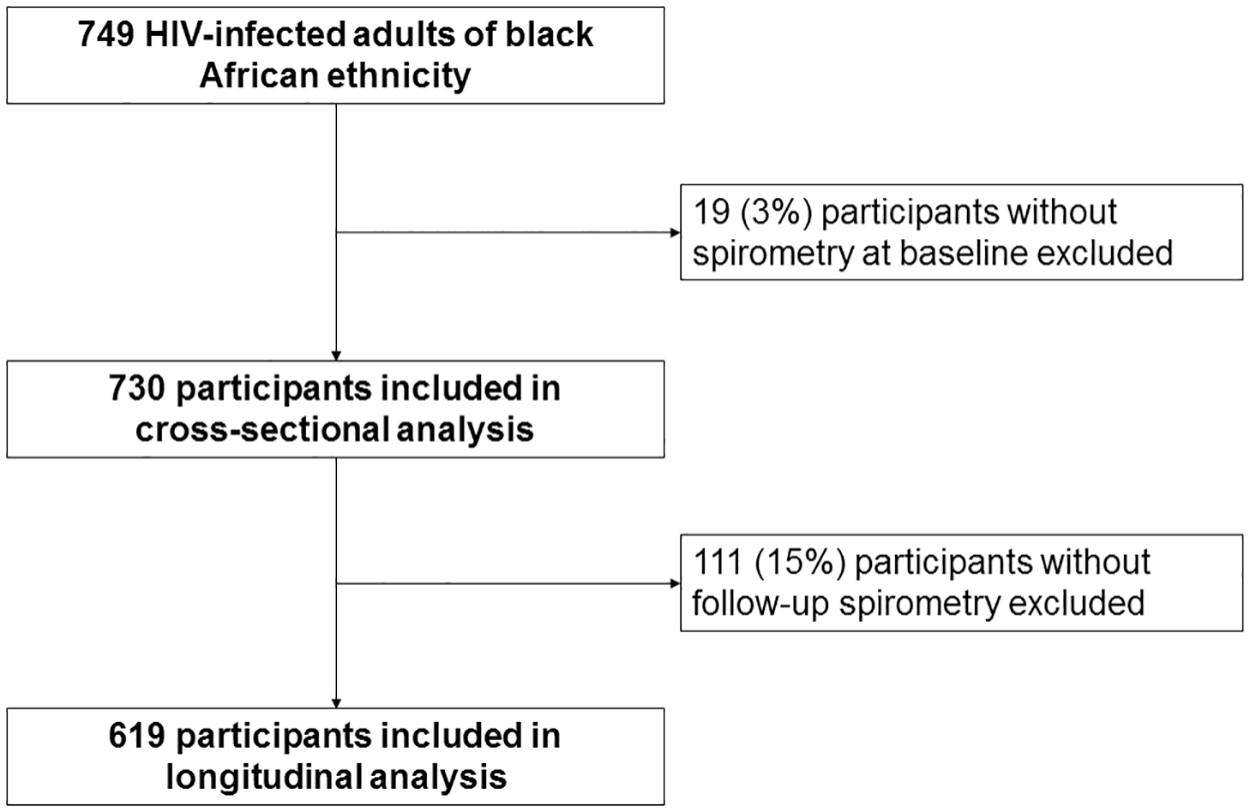
Consort diagram describing participant recruitment, exclusions and analytic sample sizes.

Of the 749 HIV-infected adults of black African ethnicity enrolled in our cohort, 19 (3%) participants did not have spirometry evaluations at enrollment and were excluded from our analysis (Fig 1). The median (IQR) age of our cohort was 36 (32–41) years and 621 (85%) were female. 220 (30%) had ever smoked; median (IQR) exposure of 3 (1–6) pack-years; and 61 (8%) were current smokers. Smoking was more common in male participants (p<0.001). A history of TB or PCP was reported by 52 (7%) and 20 (3%) respectively. Seven (13%) participants had at least two prior episodes of TB and only one participant was diagnosed with TB at enrollment. The mean (SD) duration since last episode of TB was 6 (±3) months. No one reported exposure to biomass fuels and only 2 (<1%) participants reported having ever worked in a mine. The median (IQR) CD4 count and viral load in our cohort was 372 (261–518) cells/mm^3^ and 2655 (91–13,548) copies/mL respectively, and 146 (22%) had an undetectable viral load (<50 copies/mL). Of the 180 (25%) participants on ART, 151 (84%) initiated therapy within 6 months prior to enrollment. The median (IQR) CRP levels were 3 (1–9) mg/L and did not differ significantly by a history of TB (5 vs 3 mg/L, p = 0.38) or current smoking (2 vs 3 mg/L, p>0.30) (Table 1). However, participants who recently (within 6 months of enrollment) initiated ART had higher CRP levels compared to those on chronic ART (6 vs 3 mg/L, p = 0.07) and ART-naïve individuals (6 vs 2.5 mg/L, p<0.001).

**Table 1:** Obstructive lung disease by participant characteristics at enrollment.

Characteristics	Full cohort (N = 730)	OLD present (N = 35)	OLD absent (N = 695)	p-value
**Age (years)**, median (IQR)	36 (32–41)	40 (35–48)	36 (32–41)	**<0.001**
**Female sex**, n (%)	621 (85)	23 (66)	598 (86)	**<0.001**
**BMI (kg/m**^**2**^**)**, mean (SD)	27.7 (6.4)	25.3 (5.6)	27.8 (6.4)	**0.02**
**Smoking**, n (%)				
Never	501 (69)	16 (46)	485 (71)	**<0.001**
Former	159 (22)	9 (26)	150 (22)	
Current	61 (8)	10 (29)	51 (7)	
**Pack-years smoked**, median (IQR)	3 (1–6)	5 (2–10)	3 (1–6)	0.06
**SHS**, n (%)	379 (52)	23 (66)	356 (51)	0.09
**TB**, n (%)	52 (7)	2 (6)	50 (7)	0.99
**PCP**, n (%)	20 (3)	0	20 (3)	0.61
**ART**, n (%)				
Never	549 (75)	25 (71)	524 (76)	0.34
Recent (< 6 months)	151 (21)	10 (29)	141 (20)	
Chronic (≥ 6 months)	29 (4)	0	29 (4)	
**CD4 (cells/mm**^**3**^**)**, median (IQR)	372 (261–518)	340 (239–565)	375 (261–516)	0.81
**Viral load (copies/mL)**, median (IQR)	2655 (91–13,548)	1978 (49–12,063)	2787 (115–13,622)	0.35
**CRP (mg/L)**, median (IQR)	3 (1–9)	7 (2–15)	3 (1–9)	**<0.001**
**FEV1 (L)**				
Absolute, mean (SD)	2.68 (0.52)	2.25 (0.67)	2.70 (0.50)	**<0.001**
% predicted, mean (SD)	102 (15)	84 (17)	103 (14)	**<0.001**
**FVC (L)**				
Absolute, mean (SD)	3.27 (0.66)	3.47 (1.04)	3.26 (0.63)	0.07
% predicted, mean (SD)	95 (13)	97 (19)	95 (13)	0.36
**FEV1/FVC**				
% predicted, mean (SD)	107 (9)	87 (6)	108 (7)	**<0.001**

N—number of participants, SD—standard deviation, IQR—interquartile range, OLD—obstructive lung disease, BMI—body mass index, SHS—second hand smoking, TB—self reported history of tuberculosis, PCP—self reported history of pneumocystis pneumonia, ART—anti-retroviral therapy, CRP—C-reactive protein, FEV1 –forced expiratory volume in the first second, FVC—forced vital capacity. P-values reported are comparing those with and without OLD by t-test, Wilcoxon rank sum test, X^2^ test or Fischer’s exact test as appropriate.

### Cross-sectional analysis

A total of 730 participants were included in the cross-sectional analysis at enrollment (Fig 1). Mean (SD) absolute and percent predicted FEV1 was 2.68 (±0.52) L and 102% (±15%), and FVC was 3.27 (±0.66) L and 95% (±13%) respectively. The mean (SD) absolute and percent predicted FEV1/FVC ratio was 0.82 (±0.06) and 107% (±9%) respectively. OLD (FEV1/FVC<0.70) was diagnosed in 35 (5%) participants and was more common in males (p<0.001) (Table 1).

Multivariate logistic regression analysis identified age (aOR = 2.08 per 10-year increase, 95%CI 1.22–3.57, p = 0.007), current smoking (aOR = 3.55 compared to never smokers, 95%CI 1.20–10.53, p = 0.02) and CRP (aOR = 1.01 per unit increase, 95%CI 1.00–1.03, p = 0.04) to be independently associated with OLD at enrollment. Additionally, we found a dose-response relationship between CRP levels, modelled categorically at clinically relevant thresholds, and OLD, with levels between 3 to 9 mg/L and >9 mg/L having an 8-fold (p = 0.05) and 11-fold (p = 0.02) increased odds of OLD compared to levels <1 mg/L respectively. We did not find a significant association of CD4 cell counts (aOR = 1.02 per 100-cell increase, 95%CI 0.85–1.22, p = 0.82) and ART (aOR = 0.57 compared to ART naïve, 95%CI 0.18–1.75, p = 0.32) with OLD in our cohort (Table 2). Similarly, we did not find significant associations between OLD and categorically modelled CD4 cell counts (aOR = 0.69 for counts 200 to 500 vs ≥500, 95%CI 0.28–1.65, p = 0.40 and aOR = 1.85 for counts ≤200 vs ≥500, 95%CI 0.53–6.39, p = 0.32). We found a non-significant association between viral load, modelled as a continuous variable, and OLD (aOR = 0.69 per log-increase, 95%CI 0.43–1.10, p = 0.12). When viral load was modelled as a categorical variable, participants with undetectable viral load (<50 copies/mL) had a statistically non-significant 3-fold increase in odds of OLD (95%CI 0.70–12, p = 0.13) compared to those with viral load in the greatest quartile (>13,548 copies/mL). However, we did not find similar associations between OLD and viral loads of 50 to 2650 (aOR = 0.89, 95%CI 0.29–2.72, p = 0.84) and 2650 to 13,548 (aOR = 0.99, 95%CI 0.30–3.28, p = 0.99) compared to participants with viral load in the greatest quartile. Participants with undetectable viral load were more likely to have recently (within 6 months of enrollment) initiated ART (72% vs <10%, p<0.001) and had significantly higher CRP levels (6 vs 3 mg/L, p<0.001) compared to those with detectable viral load. Odds of OLD did not differ significantly by a history of TB (aOR = 0.58, 95%CI 0.11–2.86, p = 0.50). None of the participants who reported a history of PCP or received ART for at least 6 months had OLD at enrollment.

**Table 2:** Odds ratios and 95% confidence intervals for obstructive lung disease, defined as FEV1/FVC<0.70, at enrollment.

Characteristics	Univariate	p-value	Multivariate	p-value
**Age**				
Per 10-year increase	2.27 (1.48–3.46)	**<0.001**	2.08 (1.22–3.57)	**0.007**
**Sex**				
Female	Ref.		Ref.	
Male	3.21 (1.54–6.67)	**0.002**	1.18 (0.42–3.33)	0.74
**BMI (kg/m**^**2**^**)**				
Per unit-increase	0.93 (0.87–0.99)	**0.02**	0.94 (0.88–1.02)	0.16
**Smoking status**				
Never	Ref.		Ref.	
Former	1.81 (0.78–4.19)	0.16	1.35 (0.50–3.67)	0.54
Current	5.94 (2.56–13.78)	**<0.001**	3.55 (1.20–10.53)	**0.02**
**Pack-years**				
Per unit-increase	1.06 (0.98–1.14)	0.12	-	-
**SHS**				
No	Ref.		Ref.	
Yes	1.82 (0.89–3.72)	0.09	1.67 (0.76–3.63)	0.19
**CD4 (cells/mm**^**3**^**)**				
Per 100-cell increase	1.04 (0.89–1.21)	0.56	1.02 (0.85–1.22)	0.82
**Viral load (copies/mL)**				
Per log-increase	0.84 (0.61–1.15)	0.29	0.69 (0.43–1.10)	0.12
**ART**				
Never	Ref.		Ref.	
Ever	1.23 (0.58–2.61)	0.58	0.57 (0.18–1.75)	0.32
**TB**				
No	Ref.		Ref.	
Yes	0.78 (0.18–3.34)	0.73	0.58 (0.11–2.86)	0.50
**CRP (mg/L)**				
Per unit-increase	1.02 (1.01–1.03)	**0.006**	1.01 (1.00–1.03)	**0.04**

BMI—body mass index, SHS—second hand smoking, ART—anti-retroviral therapy, TB—self-reported history of tuberculosis, CRP—C-reactive protein, Ref—reference group. Numbers in parenthesis are 95% confidence intervals. Multivariate models include age, sex, education, BMI, smoking, SHS, CD4 cell count, viral load, ART use, history of TB and plasma CRP levels. Pack-years smoked were excluded from the multivariate model due to collinearity.

We separately analyzed factors associated with OLD defined as FEV1/FVC<5^th^ percentile of expected. In addition to the 35 participants with FEV1/FVC<0.70, the percentile definition detected 22 new participants with OLD (N = 57). Consistent with our previous analysis, we did not find significant associations between CD4 cell counts, viral load or ART and OLD at enrollment. While CRP levels, modelled as a continuous variable, were not associated with OLD, we did find a marginally significant association at CRP levels above 9 mg/L (aOR = 2.55, 95%CI 0.97–6.76, p = 0.05) (S1 Table).

### Longitudinal analysis

**Fig 2:**
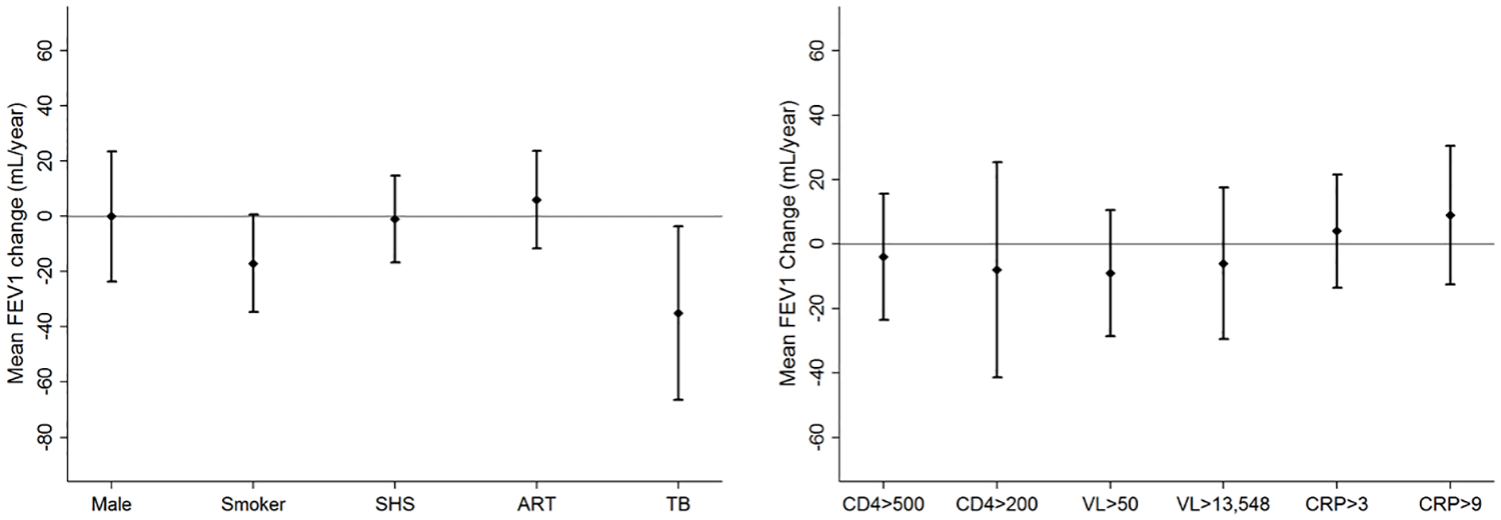
Adjusted annual excess change in FEV1 among participants with at least one follow up visit. (A) Change in mean FEV1 and accompanying 95%CI by *baseline* exposures. (B) Change in mean FEV1 and accompanying 95%CI by *time-varying* exposures. FEV1 –forced expiratory volume in 1^st^ second, CI—confidence interval, SHS—second hand smoking, ART—anti-retroviral therapy, TB—tuberculosis, CD4 –cells/mm^3^, VL—viral load in copies/mL, CRP—C reactive protein in mg/L. Excess change in FEV1 modelled as the interaction term of follow-up time and independent variable using random effects regression. Multivariate regression model includes baseline fixed variables of age, BMI, sex, education, smoking, SHS, ART and history of TB and PCP, and time-updated CD4 cell count, viral load and plasma CRP levels. Model also adjusts for baseline FEV1.

**Fig 3:**
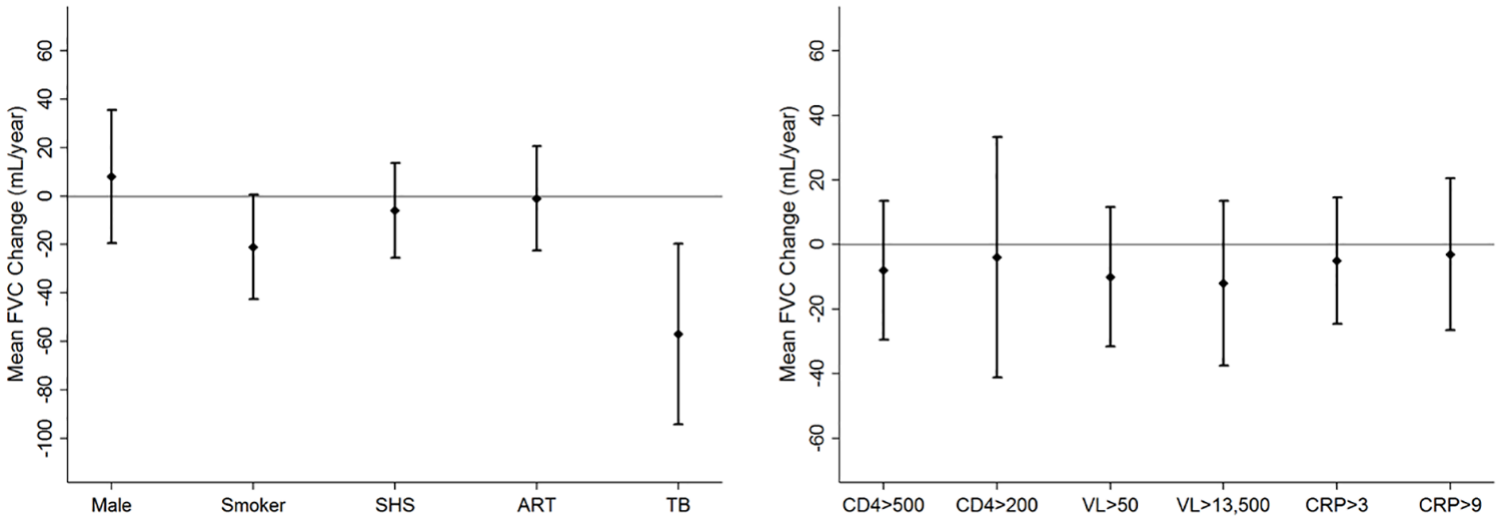
Adjusted annual excess change in FVC among participants with at least one follow up visit. (A) Change in mean FVC and accompanying 95%CI by *baseline* exposures. (B) Change in mean FVC and accompanying 95%CI by *time-varying* exposures. FVC—forced vital capacity, CI—confidence interval, SHS—second hand smoking, ART—anti-retroviral therapy, TB—tuberculosis, CD4 –cells/mm^3^, VL—viral load in copies/mL, CRP—C reactive protein in mg/L. Excess change in FVC modelled as the interaction term of follow-up time and independent variable using random effects regression. Multivariate regression model includes baseline fixed variables of age, BMI, sex, education, smoking, SHS, ART and history of TB and PCP, and time-updated CD4 cell count, viral load and plasma CRP levels. Model also adjusts for baseline FVC.

We evaluated 1705 spirometry tests over a median (IQR) follow-up time of 18 (12–24) months. Failure to complete all three years of follow-up was associated with smoking (p = 0.04), higher viral load (p = 0.02) and no ART (p = 0.01), but not OLD (p = 0.21) or prior TB (p = 0.83), at enrollment (S2 Table). We excluded 111 (15%) participants who did not have any follow-up spirometry evaluations from our analysis (Fig 1). The average FEV1 and FVC decline in our cohort was -7 mL/year (95%CI -15 to 1 mL/year, p = 0.08) and -14 mL/year (95%CI -24 to -5 mL/year, p = 0.003) respectively. Smokers had a 17 mL/year (95%CI -36 to 1 mL/year, p = 0.06) and 21 mL/year (95%CI -44 to 0 mL/year, p = 0.05) excess decline in FEV1 and FVC respectively compared to never smokers at enrollment. Participants with a history of TB at enrollment had a 35 mL/year (95%CI -68 to -2 mL/year, p = 0.03) and 57 mL/year (95%CI -96 to -19 mL/year, p = 0.003) excess decline in FEV1 and FVC respectively compared to those who did not report prior TB. Incident TB was diagnosed in 33 (5%) participants during follow up, and the association between a history of TB prior to enrollment and excess lung function decline was consistent in participants who did not develop incident TB. Overall, 157 (22%) participants initiated ART following enrollment. We did not find significant associations between ART, time-updated CD4 cell counts and viral load, and excess loss of FEV1 or FVC. While CRP levels were not associated with excess loss of FEV1, individuals with time-updated CRP of 1 mg/L or higher had a 25 mL/year excess loss of FVC (95%CI -49 to -1 mL/year, p = 0.03) (Figs 2 and 3). Additionally, we did not find significant associations between time-lagged CD4 cell counts, viral load and CRP levels, dichotomized at various thresholds, and excess loss of FEV1 or FVC (S3 Table).

Finally, we performed sensitivity analysis on 105 (17%) participants who completed all 3 years of follow-up spirometry. The average FEV1 and FVC decline in this sub-group was -21 (95%CI -34 to -8 mL/year, p<0.001) mL/year and -24 (95%CI -40 to -8 mL/year, p = 0.003) mL/year respectively. Consistent with our full cohort analysis, ever smoking and a history of prior TB at enrollment; but not time-updated CD4 cell counts and viral load, and ART use at enrollment; were significantly associated with an excess loss of FEV1. Similarly, ART receipt during follow-up among ART naïve participants at enrollment was not associated with significant differences in lung function trajectories. Interestingly, participants with a time-updated viral load of at least 13,548 copies/mL (corresponding to the highest quartile in our cohort) had a 40 mL/year (95%CI -82 to 1 mL/year, p = 0.05) excess loss of FEV1 compared to those with viral loads less than 13,548 copies/mL (S4 Table).

## Discussion

To our knowledge, our study is among the first to evaluate OLD and lung function decline in a well characterized prospective cohort of HIV-infected adults from a resource-limited setting. We found that older age, current smoking and higher CRP levels were significantly associated with prevalent OLD. Furthermore, individuals who smoked or reported a history of TB had excess lung function loss during follow up. We found a trend toward an association of OLD and viral load in our cohort, but this was not statistically significant despite our large sample size.

OLD (FEV1/FVC<0.70) was diagnosed in 5% of our cohort at enrollment. We identified 22 additional cases of OLD with FEV1/FVC<5^th^ percentile of expected yielding a prevalence of 8%. While our estimates are lower than those reported elsewhere [5, 8, 11], they are comparable to estimates from HIV-infected populations with similar smoking exposures [10, 21, 22]. The fixed ratio definition of FEV1/FVC<0.70 for OLD has been shown to underestimate airflow obstruction in younger individuals relative to a percentile approach [23], which may explain a higher diagnostic yield by the percentile approach in our relatively young cohort. Irrespective of the definition used, our estimates are comparable to the prevalence of OLD from the general population in South Africa [24].

Smoking is a well-established risk factor for excess lung function decline and airflow obstruction in HIV-uninfected individuals [25–29]. Our study extends these findings to HIV-infected adults. In our cohort, smoking was associated with a 3-fold higher odds of OLD and 17 to 21 mL/year excess decline in lung function. While it is unclear why current smoking, but not former smoking, was associated with OLD, a significantly lower pack-year exposure in former compared to current smokers (p<0.001) may explain these findings. Furthermore, our findings are consistent with those reported from studies with comparable HIV-infected populations from high-income as well as resource-limited settings [22, 30]. Burrows and colleagues found that smokers with at least 20 pack-years of exposure had significant FEV1 impairment [26]. Subsequent studies have replicated these findings by demonstrating a consistent relationship between pack-years of smoking exposure and pulmonary impairment [31–33]. However, the median pack-year exposure in our cohort was 3 (IQR 1 to 6) and only 2% reported exposures exceeding 20 pack-years. Further, none of the smokers at enrollment quit smoking (defined as no regular smoking during the preceding 6 months) during follow up. Even modest smoking was associated with pulmonary impairment in our cohort; highlighting the importance of smoking cessation in promoting lung health of HIV-infected individuals who may already have increased risk of opportunistic respiratory infections and OLD.

We found a significant association between higher CRP levels and OLD in our cohort, with the strength of association increasing at higher clinical thresholds. However, we did not find a similar consistent association between baseline or time-updated CRP levels and excess lung function decline in the prospective analysis. Discerning temporality of the association between CRP levels and OLD is a matter of considerable debate. While some studies have found a temporal association between CRP and accelerated lung function decline [34, 35], this finding may be confounded by a state of elevated systemic inflammation commonly seen in advanced COPD [36, 37]. It is possible that the association between CRP and OLD at enrollment may be secondary to immune reconstitution, which may explain higher CRP levels among participants who recently initiated ART in our cohort (median levels 6 mg/L in recent ART vs 3 mg/L in chronic ART, p = 0.07). Additionally, CRP may be elevated in commonly occurring co-morbid conditions in HIV and among smokers. While we could not adjust for duration of ART in our analysis, we did account for smoking and common co-morbid conditions such as TB that may have led to elevated CRP levels. Furthermore, the poor specificity of CRP and temporality of its association with OLD notwithstanding, we found a consistent association between high CRP levels and OLD in our cohort that was clinically relevant at thresholds known to predispose to other chronic conditions [38, 39]. Baseline CRP levels have been shown to predict incident COPD in smokers [40], and further research into the utility of CRP as a screening tool for HIV-infected individuals most likely to have OLD is recommended.

Large cross-sectional studies from multiple countries have identified an independent association between a history of TB and OLD [41–45]. Additionally, retrospective studies have found a history of TB to be associated with excess loss of lung function [46–48]. Our study is among the first to prospectively assess excess lung function decline in HIV-infected individuals with a history of TB. We found that TB, despite being treated, was associated with a 35 mL and 57 mL excess annual loss of FEV1 and FVC respectively. These estimates are comparable to excess lung function loss in smokers [31, 49, 50], highlighting the importance of chronic pulmonary impairment associated with treated TB [51], and the role of TB preventive therapy in HIV infected individuals. Furthermore, majority of participants in our cohort reporting a history of TB were recently treated prior to enrollment (range 1 to 12 months). Given the enormous burden of TB in HIV-infected individuals and its consequences to lung health, studies evaluating the role of potential host-directed adjuvant therapies to limit lung injury [52, 53] and incorporating pulmonary function assessments into routine clinical care of treated HIV-TB co-infected individuals should be considered. Surprisingly, we did not find an association between a history of TB and OLD at enrollment. TB has also been associated with lung parenchymal fibrosis and a restrictive pattern on spirometry [54–56]. Additionally, our longitudinal analysis found a higher FVC loss relative to FEV1 among those with a history of TB. A predominantly restrictive phenotype of lung functional impairment among participants reporting a history of TB may explain the lack of a significant TB-OLD association in our cohort.

While our findings differ from those reported from high-income settings, they are similar to those reported from a recent multi-center study of HIV-infected adults with comparable demographic and clinical characteristics [22]. Contrary to what was reported in the VA study [5], we did not find a significant association between CD4 cell counts and OLD in our cohort. While the precise reasons for these differences are unclear, differences in population characteristics (older males vs predominantly younger females), risk behaviors (heavy smoking vs low smoking exposure), study setting (high income vs resource limited country) and different outcome definitions (ICD-9 vs spirometry-based) may explain some of the inconsistencies. Similarly, very high viral load was associated with prevalent OLD, and poorly controlled HIV disease (high viral load with or without low CD4 cell counts) resulted in an excess loss of lung function in the ALIVE cohort [8, 9]. We did not find similar associations in our cohort. Our study population differs from that of the ALIVE cohort in terms of heterogeneity of disease severity, and potential confounders such as smoking and IDU. For instance, the viral load cut-point for identifying the highest quartile in the ALIVE cohort was 30,700 copies/mL compared to 13,548 copies/mL in our study. Furthermore, 26% of the ALIVE cohort participants ever had a viral load above 200,000 copies/mL; the threshold for a significant association with prevalent OLD in the study; compared to only 2% of our study population. Limited number of individuals with poorly controlled HIV disease may explain the lack of significant associations between viral load and OLD or excess lung function decline in our study. We may also be underpowered to detect a true effect of viral load on excess lung function decline due to a high attrition rate in our cohort; which may explain a marginally significant 40mL/year excess decline in FEV1 among individuals in the highest quartile of viral load in our sensitivity analysis restricted to participants who completed all study visits.

Another possible explanation for these differences may be the effect of smoking on HIV-associated lung injury. Airflow obstruction in smokers is primarily CD8 T cell mediated, and is associated with cellular infiltration and alveolitis [57]. This mechanism is strikingly similar to what has been proposed for HIV-associated pulmonary impairment [58–60]. It is possible that the deleterious effect of HIV on the lungs may be modified, or even triggered, by smoking exposure, with accelerated lung function decline manifesting primarily in individuals with both high levels of smoking and viral load exposures. This may explain the lack of similar associations in our cohort of relatively well controlled HIV disease and low smoking exposure. Due to the absence of HIV-uninfected individuals in our cohort, we could not test for interaction between HIV and smoking, and given the clinical implications of HIV associated lung injury, this hypothesis warrants further investigation.

Current evidence on the association between ART and OLD in adults is conflicting. Oxidative stress, immune reconstitution and autoimmune reactions have been proposed as possible mechanisms for ART associated lung injury [61]; however, the precise mechanisms are unclear. Two recent studies found a significant association between ART and prevalent OLD [10, 11]. However, these findings were not replicated in subsequent studies [8, 12, 13] and ART had a protective effect against incident COPD in the VA study [7]. We did not find a significant association between ART and prevalent OLD or excess lung function decline in our cohort. Nearly 75% of our cohort was ART naïve at enrollment and majority of participants who ever received ART did so within the 6 months prior to enrollment. While ART initiation was primarily driven by CD4 cell counts, majority of participants with undetectable viral load or high CRP levels at enrollment had recently (within 6 months) initiated ART. Despite relatively low levels of immune suppression in our cohort, inflammatory lung injury secondary to rapid immune reconstitution could explain the trend towards higher odds of OLD among participants with undetectable viral load or higher CRP levels in our cohort [61, 62]. Further research to establish a causal link, if any, between ART and pulmonary impairment is needed.

Our study has limitations. Post-bronchodilator spirometry was not performed in our cohort and we could not test for reversibility of airflow obstruction. Second, the cross-sectional nature of our primary analysis precludes the identification of a temporal relationship between host factors and prevalent OLD. However, with the exception of CRP, cross-sectional findings are consistent with those from our longitudinal analysis. Third, a history of TB was assessed by self-report and may be subject to recall bias or misclassification. However, this cohort was recruited from a clinical trial where TB was rigorously assessed [15] and from a patient care cohort where symptoms of TB were rigorously investigated. Fourth, the date of HIV diagnosis was not available in our cohort. Duration of HIV exposure may be an important risk factor for pulmonary impairment. However, the precise duration of HIV infection is rarely known and years lived with HIV was not associated with OLD elsewhere [11]. Further, current understanding of the mechanism driving HIV associated lung injury suggests a role of pulmonary inflammation secondary to high viral load or immune reconstitution. The biologically relevant window of exposure may therefore be unrelated to the duration of HIV infection. Finally, our secondary analysis exploring factors associated with excess declines in lung function may be underpowered due to a high attrition rate that was more common in participants with higher viral loads. Our findings may therefore have limited generalizability to individuals with poorly controlled HIV disease.

Despite these limitations our study is among the first to report on factors associated with OLD and excess lung function decline in HIV-infected individuals prospectively evaluated from a well characterized cohort in a high HIV-TB burden setting. In contrast to published work, our findings extend our understanding of HIV associated pulmonary impairment by studying a cohort of relatively well controlled HIV disease with minimal exposure to confounders such as smoking, IDU, biomass fuel use and occupation. Our findings are likely generalizable to HIV-infected individuals receiving active clinical care in resource limited settings, who account for a vast majority of the HIV burden globally. In conclusion, prevalent OLD was associated with older age, current smoking and higher CRP levels; but not CD4 count and ART; in HIV-infected South African adults. Better understanding of the long-term effects of TB, smoking and inflammation on lung function in HIV-infected populations is urgently needed.

## Supporting information

S1 Table Odds ratios and 95% confidence intervals for obstructive lung disease, defined as FEV1/FVC<5th percentile of expected, at enrollment (N = 730). 57 (8%) participants had FEV1/FVC<5^th^ percentile of expected at enrollment.
(DOCX)

S2 Table Baseline characteristics of participants with and without three years of follow-up. (DOCX)

S3 Table Adjusted annual excess change in lung function by time-lagged CD4 cell count, viral load and CRP levels (N = 619). (DOCX)

S4 Table Adjusted annual excess change in lung function by baseline and time-varying exposures among participants with three years of follow-up (N = 105). (DOCX)
