# Supplementary material for: Factors associated with pulmonary impairment in HIV-infected South African adults
Source: PLoS One. 2017 Sep 13;12(9):e0184530. doi: 10.1371/journal.pone.0184530 (PMC5597201; doi:10.1371/journal.pone.0184530)
Supplement: S1 Table — 57 (8%) participants had FEV1/FVC<5th percentile of expected at enrollment. (DOCX) [file pone.0184530.s001.docx]

**S1 Table. Odds ratios and 95% confidence intervals for obstructive lung disease, defined as FEV1/FVC<5th percentile of expected, at enrollment (N=730).** 57 (8%) participants had FEV1/FVC<5^th^ percentile of expected at enrollment.

| **Characteristics** | **Univariate** | **p-value** | **Multivariate** | **p-value** |
| --- | --- | --- | --- | --- |
| **Age** |  |  |  |  |
| Per 10-year increase | 1.27 (0.88-1.83) | 0.19 | 1.05 (0.66-1.67) | 0.83 |
| **Sex** |  |  |  |  |
| Female | Ref. |  | Ref. |  |
| Male | 4.65 (2.61-8.28) | **<0.001** | 2.22 (0.98-5.01) | **0.05** |
| **BMI (kg/m^2^)** |  |  |  |  |
| Per unit-increase | 0.90 (0.86-0.95) | **<0.001** | 0.94 (0.88-1.00) | 0.06 |
| **Smoking status** |  |  |  |  |
| Never | Ref. |  | Ref. |  |
| Former | 2.46 (1.30-4.64) | **0.005** | 1.47 (0.66-3.25) | 0.33 |
| Current | 5.65 (2.75-11.62) | **<0.001** | 2.82 (1.14-6.94) | **0.02** |
| **Pack-years** |  |  |  |  |
| Per unit-increase | 1.06 (0.99-1.13) | 0.06 | - | - |
| **SHS** |  |  |  |  |
| No | Ref. |  | Ref. |  |
| Yes | 1.80 (1.02-3.17) | **0.04** | 1.50 (0.80-2.91) | 0.19 |
| **CD4 (cells/mm^3^)** |  |  |  |  |
| Per 100-cell increase | 0.99 (0.87-1.13) | 0.91 | 1.00 (0.86-1.16) | 0.96 |
| **Viral load (copies/mL)** |  |  |  |  |
| Per log-increase | 1.05 (0.81-1.35) | 0.69 | 0.81 (0.56-1.16) | 0.26 |
| **ART** |  |  |  |  |
| Never | Ref. |  | Ref. |  |
| Ever | 0.70 (0.35-1.40) | 0.32 | 0.51 (0.19-1.31) | 0.16 |
| **TB** |  |  |  |  |
| No | Ref. |  | Ref. |  |
| Yes | 0.97 (0.33-2.81) | 0.96 | 1.01 (0.32-3.15) | 0.98 |
| **CRP (mg/L)** |  |  |  |  |
| Per unit-increase | 1.01 (0.99-1.02) | 0.08 | 1.00 (0.99-1.02) | 0.19 |
| N – number of participants, OLD – obstructive lung disease defined as FEV1/FVC<5^th^ percentile of expected, BMI – body mass index, SHS – second hand smoking, ART – anti-retroviral therapy, TB – self-reported history of tuberculosis, CRP – C-reactive protein, Ref – reference group.  Numbers in parenthesis are 95% confidence intervals.  Multivariate models include age, sex, education, BMI, smoking, SHS, CD4 cell count, viral load, ART use, history of TB and plasma CRP levels. Pack-years smoked were excluded from the multivariate model due to collinearity.  None of the participants who reported a history of PCP or at least 6 months of ART had OLD at enrollment.  Age, sex and height expected FEV1 and FVC was calculated from prediction equations by Mokoetle and colleagues for adults of black African ethnicity residing in Johannesburg. | | | | |
