# Supplementary material for: Factors associated with pulmonary impairment in HIV-infected South African adults
Source: PLoS One. 2017 Sep 13;12(9):e0184530. doi: 10.1371/journal.pone.0184530 (PMC5597201; doi:10.1371/journal.pone.0184530)
Supplement: S2 Table — (DOCX) [file pone.0184530.s002.docx]

**S2 Table. Baseline characteristics of participants with and without three years of follow-up.**

| **Characteristics** | **3 years of follow-up (N=105)** | **< 3 years of follow-up (N=625)** | **p-value** |
| --- | --- | --- | --- |
| **Age (years)**, median (IQR) | 37 (32-41) | 36 (32-41) | 0.19 |
| **Female sex**, n (%) | 91 (87) | 530 (85) | 0.76 |
| **BMI (kg/m^2^)**, mean (SD) | 28.6 (7.1) | 27.6 (6.2) | 0.11 |
| **Smoking**, n (%) |  |  |  |
| Never | 80 (78) | 421 (68) | **0.04** |
| Former | 20 (19) | 139 (22) |  |
| Current | 3 (3) | 58 (9) |  |
| **Pack-years smoked**, median (IQR) | 6 (2-10) | 3 (1-6) | 0.07 |
| **Second-hand smoking**, n (%) | 52 (50) | 326 (52) | 0.75 |
| **TB**, n (%) | 8 (8) | 44 (7) | 0.83 |
| **PCP**, n (%) | 0 | 20 (3) | 0.09 |
| **ART**, n (%) | 37 (35) | 143 (23) | **0.01** |
| **CD4 (cells/mm^3^)**, median (IQR) | 360 (258-505) | 375 (262-527) | 0.78 |
| **Viral load (copies/mL)**, median (IQR) | 1742 (49-8325) | 2952 (169-14,823) | **0.02** |
| **CRP (mg/L)**, median (IQR) | 4.5 (1-10) | 3 (1-9) | 0.34 |
| **% predicted FEV1** **(L)**, mean (SD) | 103 (15) | 102 (15) | 0.42 |
| **% predicted FVC (L)**, mean (SD) | 94 (14) | 95 (13) | 0.47 |
| **OLD**, n (%) | 2 (2) | 33 (5) | 0.21 |
| N – number of participants, SD – standard deviation, IQR – interquartile range, OLD – obstructive lung disease, BMI – body mass index, TB – self reported history of tuberculosis, PCP – self reported history of pneumocystis pneumonia, ART – anti-retroviral therapy, CRP – C-reactive protein, FEV1 – forced expiratory volume in the first second, FVC – forced vital capacity.  P-values reported are comparing those with and without OLD by t-test, Wilcoxon rank sum test, X^2^ test or Fischer’s exact test as appropriate. | | | |
