# Supplementary material for: Factors associated with pulmonary impairment in HIV-infected South African adults
Source: PLoS One. 2017 Sep 13;12(9):e0184530. doi: 10.1371/journal.pone.0184530 (PMC5597201; doi:10.1371/journal.pone.0184530)
Supplement: S3 Table — (DOCX) [file pone.0184530.s003.docx]

**S3 Table.** **Adjusted annual excess change in lung function by time-lagged CD4 cell count, viral load and CRP levels (N=619).**

| **Time-lagged exposure** | **∆ FEV1 (95%CI)**  **mL/year** | **p-value** | **∆ FVC (95%CI)**  **mL/year** | **p-value** |
| --- | --- | --- | --- | --- |
| **CD4 (cells/mL)** |  |  |  |  |
| >500 vs < 500 | -2 (-34 to 29) | 0.87 | -20 (-56 to 15) | 0.27 |
| >350 vs < 350 | -10 (-42 to 20) | 0.48 | -17 (-51 to 16) | 0.32 |
| >200 vs < 200 | -41 (-101 to 18) | 0.17 | -47 (-115 to 20) | 0.17 |
| **Viral load (copies/mL)** |  |  |  |  |
| >50 vs < 50 | 4 (-26 to 36) | 0.76 | 3 (-31 to 37) | 0.86 |
| >200 vs < 200 | 0 (-28 to 27) | 0.96 | 7 (-22 to 38) | 0.60 |
| >13,548 vs < 13,548 | 8 (-30 to 47) | 0.65 | 1 (-41 to 43) | 0.95 |
| **CRP (mg/L)** |  |  |  |  |
| >1 vs <1 | 1 (-32 to 34) | 0.94 | -3 (-40 to 34) | 0.86 |
| >3 vs < 3 | -2 (-31 to 26) | 0.88 | -3 (-36 to 28) | 0.81 |
| >9 vs < 9 | 0 (-37 to 37) | 0.99 | -9 (-51 to 32) | 0.65 |
| FEV1 – forced expiratory volume in 1^st^ second, FVC – forced vital capacity, CI – confidence interval, SHS – second hand smoking, ART – anti-retroviral therapy, TB – tuberculosis, PCP – pneumocystis pneumonia, CRP – C-reactive protein.  ∆ – Excess change modelled as the interaction term of follow-up time and independent variable using random effects regression.  Multivariate regression models include baseline fixed variables of age, BMI, sex, education, smoking (ever vs never), SHS, ART and history of TB and PCP, and time-lagged CD4 cell count, viral load and plasma CRP levels. Models estimating excess annual decline in FEV1 and FVC also adjust for baseline FEV1 and FVC respectively. | | | | |
